# Lung Cancer Nodules Detection via an Adaptive Boosting Algorithm Based on Self-Normalized Multiview Convolutional Neural Network

**DOI:** 10.1155/2022/5682451

**Published:** 2022-09-26

**Authors:** Adeel Khan, Irfan Tariq, Haroon Khan, Sifat Ullah Khan, Nongyue He, Li Zhiyang, Faisal Raza

**Affiliations:** ^1^State Key Laboratory of Bioelectronics, School of Biological Science and Medical Engineering, Southeast University, Nanjing, China; ^2^Department of Biotechnology, University of Science and Technology, Bannu, KP, Pakistan; ^3^School of Information Science and Engineering, Southeast University, Nanjing, China; ^4^Neuroscience and Neuroengineering Research Center, Med-X Research Institute, School of Biomedical Engineering, Shanghai Jiao Tong University, Shanghai 200240, China; ^5^School of Electronics Science and Engineering, Southeast University, Nanjing, China; ^6^Department of Clinical Laboratory, Nanjing Drum Tower Hospital Affiliated with Nanjing University Medical School, Nanjing, China; ^7^School of Pharmacy, Shanghai Jiao Tong University, Shanghai, China

## Abstract

Lung cancer is the deadliest cancer killing almost 1.8 million people in 2020. The new cases are expanding alarmingly. Early lung cancer manifests itself in the form of nodules in the lungs. One of the most widely used techniques for both lung cancer early and noninvasive diagnosis is computed tomography (CT). However, the intensive workload of radiologists to read a large number of scans for nodules detection gives rise to issues like false detection and missed detection. To overcome these issues, we proposed an innovative strategy titled adaptive boosting self-normalized multiview convolution neural network (AdaBoost-SNMV-CNN) for lung cancer nodules detection across CT scans. In AdaBoost-SNMV-CNN, MV-CNN function as a baseline learner while the scaled exponential linear unit (SELU) activation function normalizes the layers by considering their neighbors' information and a special drop-out technique (*α*-dropout). The proposed method was trained and tested using the widely Lung Image Database Consortium and Image Database Resource Initiative (LIDC-IDRI) and Early Lung Cancer Action Program (ELCAP) datasets. AdaBoost-SNMV-CNN achieved an accuracy of 92%, sensitivity of 93%, and specificity of 92% for lung nodules detection on the LIDC-IDRI dataset. Meanwhile, on the ELCAP dataset, the accuracy for detecting lung nodules was 99%, sensitivity 100%, and specificity 98%. AdaBoost-SNMV-CNN outperformed the majority of the model in accuracy, sensitivity, and specificity. The multiviews confer the model's good generalization and learning ability for diverse features of lung nodules, the model architecture is simple, and has a minimal computational time of around 10^2^ minutes. We believe that AdaBoost-SNMV-CNN has good accuracy for the detection of lung nodules and anticipate its potential application in the noninvasive clinical diagnosis of lung cancer. This model can be of good assistance to the radiologist and will be of interest to researchers involved in the designing and development of advanced systems for the detection of lung nodules to accomplish the goal of noninvasive diagnosis of lung cancer.

## 1. Introduction

**Figure 1:**
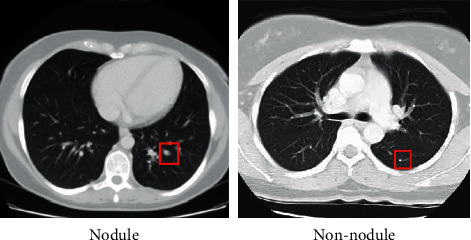
(a) Nodule and (b) nonnodule.

Lung cancer is the deadliest cancer in terms of death rates across the globe [1]. In the year 2020, lung cancer has been devastating for humanity, causing the death of 1.80 million individuals and 2.21 million new cases. By 2040, the number of new cases is projected to be 3.63 million, while the number of deaths to be 3.01 million (Global Cancer Observatory, World Health Organization). The main concern is the stealth progression of lung cancer and patients are mostly diagnosed at the advanced stage (metastatic stage) with fewer chances of survival [2]. Therefore, many efforts are underway to develop tools and techniques to accomplish noninvasive and early diagnosis of lung cancer [3]. Computed tomography (CT) is the most widely used robust and noninvasive technique to screen for lung cancer diagnosis [4]. CT scans are capable of detecting the smallest tumors. The smaller the tumor, the better the chances of treatment [5]. A 20% decrease in lung cancer deaths has been observed in high-risk individuals when screened via CT annually [6]. The detection of lung nodules is critical for lung cancer diagnosis, as they are the most important radiological indicator to accomplish early detection of lung cancer. The size of the nodules has a direct relation with the state of malignancy. Lung nodules in most cases can be a miniature form of lung tumors [7]. However, there is a high degree of heterogeneity in their sizes, shapes, and types, therefore, the efficient detection of lung nodules is a challenging task [8]. Some guidelines provide information about nodule size and its relationship with the rate of malignancy. It has been reported that if the diameter of nodules is less than 5 mm, the chances of malignancy are 1%. If the diameter is between 6 mm and 10 mm, the chances of malignancy are about 24%. Nodules with a diameter between 11 mm and 20 mm suggest 33% chance of malignancy; if the diameter exceeds 20 mm, the chances of malignancy are about 80% [9]. The above facts clearly indicate the importance of nodule detection in the diagnosis of lung cancer [10]. Nowadays CT scans are widely recommended, therefore, the workload of radiologists is exacerbated. Manual detection of nodules by analyzing CT scans is a toilsome task and mistake prone; therefore, to assist computer-aided diagnosis (CAD) can be valuable for nodule detection that will not only lessen the workload of radiologists but will also improve the diagnostic precision by only taking up short time [11]. CAD techniques have shown incredible prospects in the field of cancer imaging. Deep learning-based approaches generate excellent results because during the training phase, the model self-learn the crucial features. Self-learning confers the model with the ability to gather information on the most crucial features of the nodule from CT scans. A highly variable training set helps the model learn the most crucial and consistent features of a nodule and hence, can accomplish improved and specific detection. After the training phase, the model can efficiently work on scans, appearing for the first time, to make decisions about nodules and nonnodule as shown in Figure 1 based on the purpose it has been trained for [12].

**Figure 2:**
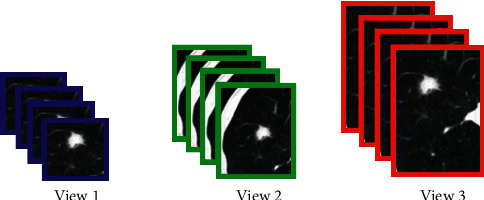
Multiviews. View 1 (20 *∗* 20), view 2 (30 *∗* 30), and view 3 (40 *∗* 40).

We designed an AdaBoost-basedself-normalized MV-CNN (SNMV-CNN) model named AdaBoost-SNMV-CNN. MV-CNNs had shown better performance for the identification of lung cancer nodules. In contrast to conventional CNN which receives single input, the MV-CNN operates by receiving multiple inputs. The underlying objective for using MV-CNN in this study is to improvise the processing of multiple views of lung nodules (different-sized cropped images or patches of nodules, as shown in Figure 2. We anticipated that views with smaller receptive fields will provide ample information about nodules while the views with the large receptive field will provide information about the surrounding areas. As a result, more information is gathered about nodules. Similarly, AdaBoost is an ensemble learning method [13, 14] and the incorporation of self-normalized MV-CNN (SNMV-CNN) in AdaBoost (AdaBoost-SNMV-CNN) enriches the model with superior learning, analyzing, and finding pattern capabilities of MV-CNN for nodules identification and the AdaBoost characteristic of dealing with large datasets; for instance, Lung Image Database Consortium and Image Database Resource Initiative (LIDC-IDRI) dataset and Early Lung Cancer Action Program (ELCAP) dataset. The reported method AdaBoost-SNMV-CNN has the transfer learning property of deep learning methods that reduces the computational cost and time making it superior to existing methods. The results are promising in terms of classification accuracy, sensitivity, specificity, and AUC with reduced false positives.

Our main contributions are as follows: (1) We proposed the incorporation of MV-CNN in an AdaBoost algorithm for lung cancer nodules detection for the first time. (2) The incorporation of MV-CNN assists in collecting more information about heterogeneous nodules. (3) AdaBoost-SNMV-CNN architecture is simple and has less computational time. (4) The model showed high accuracy, sensitivity, and specificity as it involves transfer learning: the knowledge acquired by the initial base learner (MV-CNN) is transferred to the next, significantly reducing training time and computational cost. (5) The proposed method was trialed using LIDC-IDRI datasets and ELCAP dataset to test its generalization ability and acquired good results compared to the majority of previous methods reported for the detection of lung cancer nodules.

The manuscript further comprises the following sections. Section 1.4 includes a brief overview of related work.  Section 2 includes the material and methods, an explanation of input datasets, and a detailed explanation of the AdaBoost-SNMV-CNN model upon which our manuscript is based.  Section 3 comprises a detailed explanation of the results.  Section 4 includes discussion, and Section 5 includes the limitations and future work.  Section 6 comprises the final conclusion of the work reported in the manuscript.

### 1.1. Related Work

Till now, around 65% of lung cancer patients are diagnosed at late stages which greatly diminishes their chances of recovery. Thus, there is an urgent need for noninvasive CAD systems that can accomplish the early diagnosis of lung cancer through CT scans [4]. The appearance of advanced deep learning algorithms with the intrinsic ability to automatically extract features from the data has made it possible to achieve excellent performance in a variety of domains including image analysis and also in nodules detection from CT images. Deep learning has shown prospects in lung nodules detection and classification [15]. Traditionally, CNN has been employed by many research groups for the purpose of lung nodule identification. Recently, researchers have proposed a new deep learning approach based on MV-CNN for the classification of lung nodules. The MV-CNN-based method outperformed the single-view methodologies [16]. Another group of researchers proposed a multiview and multiscale CNN for the distinction of nodules and nonnodules [17]. Similarly, to distinguish nodules, a multistream and multiscale CNN was employed that achieved a performance level comparable to the human level [18]. Researchers are now employing 2D and 3D CNN-based approaches for lung nodules detection. 3D CNN-based approaches are preferred over 2D CNN-based approaches as lung nodules detection is actually a 3D object detection, and also 3D CNN-based approaches consider volumetric information for lung nodules detection [19]. A research group proposed a 3D MV-CNN-based approach for the classification of lung cancer nodules and also demonstrated that 3D MV-CNN performance is better than the 2D MV-CNN [16]. A multilevel contextual 3D CNNs can accurately distinguish true lung nodules from candidates and significantly reduces the false-positive rate. 3D CNN learns more features and collects more spatial information. The integration of multilevel contextual information makes the model more robust. The model performance on LIDC-IDRI dataset was robust and best [20]. However, 3D CNN has limitations, such as increased parameter freedom, that may cause the model to overfit on training data. The size of the model is big, so consumes more time and memory. There is also a scarcity of annotated 3D data to be used for training 3D CNN-based models [21].

## 2. Materials and Methods

### 2.1. Datasets

#### 2.1.1. LIDC-IDRI

The Lung Image Database Consortium and Image Database Resource Initiative (LIDC-IDRI) has 1018 CT scans in DICOM format with marked-up labeled lesions. In XML format, diagnostic annotations are offered. Four thoracic radiologists completed a two-stage annotation and revision protocol on each CT scan. The radiologists independently analyzed each CT image in the first phase and identified lesions status (“nodule ≥3 mm,” “nodule <3 mm,” and “non-nodule ≥3 mm”). After that, each radiologist examined the labels of the remaining lesions independently and presented their confirmed diagnosis [22]. Selection of candidate positions and data generation for model development were accomplished using a published methodology [21]. A total of 2370 candidate positions were acquired (1185 nodules and 1185 nonnodules). Totally 14,220 images (7110 2D images, 7110 3D images) were used in model development. The training and testing data followed the 80/20 division rule, respectively.

#### 2.1.2. ELCAP

ELCAP dataset (Accessed January 22, 2022, https://www.via.cornell.edu/lungdb.html) is the result of a collaborative effort of Early Lung cancer Action Program (ELCAP) and Vision and Image Analysis Group (VIA). The effectiveness of CAD systems is mostly evaluated using this dataset. The dataset comprises 50 low-dose CT scans of 1.25 mm slice thickness for a single breath hold for the purpose of lung cancer detection. Radiologists have only annotated the nodules' locations. Following [21] methodology, we considered around 902 candidate positions (451 nodules and 451 nonnodules). As the ELCAP dataset contains only nodules positions, the requirement of nonnodules candidates was met by including the nonnodules locations from the LIDC-IDRI dataset. The inclusion of nonnodules positions made the dataset suitable for binary classification problems and also made it possible to expose the model to learn generalized details about nodules and nonnodules. The model was exposed to around 5412 images divided through the 80/20 rule for training and testing, respectively.

### 2.2. AdaBoost

The AdaBoost algorithm is a short form of adaptive boosting introduced by [23]. AdaBoost is an ensemble method; so, the sequence of base learners (as in our proposed methods MV-CNNs) is trained. Each training input has been assigned a weight; more weight is assigned to the training input that has not been trained by the prior learner. This weight adjustment directs the proposed model to pay more attention to the error in the next round; this significantly reduces the problem of misidentification or missing identification.

### 2.3. Multiview CNN (MV-CNN)

MV-CNN is an advanced form of CNN; differing in a way that MV-CNN is exposed to multiview of the same input, making them exhibit superior learning performance than the traditional CNN. A CNN module is composed of convolution and pooling layers. The convolutional layers perform the operation of feature extraction from the images [16, 21]. The convolution operation is denoted as:

(1) $$\begin{array}{c} f_{k}\left(x,y\right)=\text{SELU}\left(b_{k}+\sum_{k}\sum_{u,v}D_{k}\left(x-u,y-v\right)*W_{k}\left(u,v\right)\right). \end{array}$$

In the above equation, the image is denoted by *D* and the filter for the *k*^*th*^ map is denoted by *W*. The “*∗*” sign represents the inner product of *D* and *W*. After the inner product, a bias term is added denoted by *b*. We also employed SELU in this study, which is a nonlinear activation function.

After the convolution layer, there is maxpooling layer that accomplishes the selection of maximum value within a receptive field [24]. Maxpooling operation follows the following equation:

(2) $$\begin{array}{c} M_{kij}=\underset{\left(s,t\right)\varepsilon DR_{i,j}}{\max}\left(X_{kst}\right). \end{array}$$

In the above equation, *M*_*kij*_ is the output of maxpooling operation for the *k*^*th*^ feature map. (*s*, *t*) denotes the location of the element (*X*_*kst*_) which is the pooling region (*R*_*i*,*j*_) that covers the receptive field around the position (*i*, *j*).

Once the features are extracted via convolutional and maxpooling layers, fully connected layers are used to combine them. The fully connected layer carries out high-level reasoning through interpretation of the features in context [25]. In the fully connected layer, denser connections are present such that each neuron is connected with all the neurons in the next-neighboring layer, as follows:

(3) $$\begin{array}{c} h^{t}=\text{SELU}\left(b^{t}+h^{t-1}W^{t}\right). \end{array}$$

In the above equation, *h*^*t*^ denotes output and *h*^*t*^ − 1 denotes an input feature vector. *W*^*t*^ represents weight matrix and *b*^*t*^ represents bias term.

### 2.4. Scaled Exponential Linear Units (SELU)

In CNN, the activation function is applied to each component of a feature map for introducing the nonlinearity in CNN. We used scaled exponential linear units (SELU) [26] as an activation function. In general, CNN use rectified linear units (ReLU) as activation. ReLU activation clips negative values to 0 and suffers from dying ReLU problem. As explained by [26], an activation function should contain both positive and negative values for controlling mean, saturation regions for reducing high variance and slope greater than one to increase variance if its value is too small. Hence, SELU activation was introduced to preserve the aforementioned properties. SELU activation function can be defined as:

(4) $$\begin{array}{c} \text{SELU}\left(x\right)=\lambda \left\{\begin{array}{cc} x, & \text{if }x>0,\\ \alpha e^{x}, & \text{if }x<0, \end{array}\right) \end{array}$$

where *x* denotes input, *α*(*α*=1.6733), *λ*(*λ*=1.0507) are hyperparameters, and *e* stands for exponent.

### 2.5. Proposed AdaBoost-SNMV-CNN

In the proposed algorithm, the self-normalized multiview convolutional neural network (SNMV-CNN) was used as a base classifier. The research community has begun to use MV-CNNs by virtue of their promising performance with respect to their counterpart single-view CNNs (SV-CNNs). The main difference between an SV-CNN and MV-CNN is their input; in MV-CNN, different views of the same input are passed to the model to learn generalized features from the data. An MV-CNN gathers more information from all the views instead of just averaging, hence can robustly detect an object such as lung cancer nodules compared to SV-CNN [16] and almost comparable to 3D CNN with advantages such as less computational time [27]. Also noted that in the proposed AdaBoost-SNMV-CNN, we can make adjustments in data input; first, find the geometrical center of each nodule, then centered on these points, crop patches in three different sizes generating multiple views. After that, we used spline interpolation to resize them into the same size before feeding them into different channels. MV-CNN is composed of an input layer, convolution layers, pooling layers, and fully connected layers. In which *w* denotes the filters and *y* is the output. Similarly, the pooling is done on convolutional layer. During the pooling, a filter moves across the convolutional output to take the average value. The goal of pooling layer is to progressively reduce the spatial size of the matrix as shown in equation ??. In which, *p* denotes the pooling size. Let the given lung cancer CT images dataset comprise of *n* training samples, *D* = {(*x*_1_, *c*_1_), (*x*_2_, *c*_2_),…, (*x*_*i*_, *c*_*i*_),…, (*x*_*n*_, *c*_*n*_)}, where *x*_*i*_ is the lung cancer CT images data input feature instance and *c*_*i*_ is the label (nodule or nonnodule). In proposed model, SNMV-CNN is used as a base learner, and fed with multiviews following [16, 21] and the output prediction of the model is denoted by *H* = {*f*_1_, *f*_2_,…, *f*_*m*_}. The purpose of training is to fit the model to a particular data and then evaluate the performance of the trained model using unseen testing data. Each sample in the training data was allocated a weight for simplicity, without disturbing the generality. Suppose that the weight distribution over these samples at the *m*^*th*^ boosting iteration is denoted by *S*_*m*_. On the first iteration, the SNMV-CNN was trained on the same data weight initialized by (1/*n*). There is no difference in importance and weight for the training samples in the first SNMV-CNN. For the particular base learner, the prediction error that arises during the training sample can be obtained using the following equation:

(5) $$\begin{array}{c} \varepsilon_{m}=\sum_{1}^{n}S_{m}\left(i\right)\times \left\{\begin{array}{cc} 0, & \text{if }f_{m}\left(x_{i}\right)=c_{i},\\ 1, & \text{if }f_{m}\left(x_{i}\right)\neq c_{i}, \end{array}\right) \end{array}$$

here *c*_*i*_ denotes an observed label for the input sample *x*_*i*_, *ε*_*m*_ denotes the classification error of the current classifier, and *S*_*m*_ denotes the trained SNMV-CNN network at iteration *m*. Based on the performance of the current learner on the training dataset, the weight is distributed in a manner that correctly detected samples are given a lower weight and incorrectly detected samples a higher weight. The equation for the process of weight updating is as follows:

(6) $$\begin{array}{c} S_{m+1}\left(i\right)=\frac{S_{m}}{Z_{m}}\exp\left(-d_{m}\times c_{i}\times f_{m}\left(x_{i}\right)\right). \end{array}$$

In the above equation, *Z*_*m*_ denotes the normalization constant ensuring the exact distribution of *S*_*m*+1_(*i*), and *d*_*m*_ denotes the voting weight for the trained classifier *f*_*m*_. The exponential function in the above equation is miniaturized when a correlation exists between the output vector and output label, and has a large internal product (due to the negative sign). The low value of the exponential function in equation (6) consequently lowers the weight of the training sample of the subsequent SNMV-CNN since the actual output is close to the label and demonstrates that the current SNMV-CNN is trained on the training samples. When the weights updating for current SNMV-CNN is completed on the training samples, the weights are normalized and divided by the total sum of the weights. The current trained SNMV-CNN is stored and the learning of a subsequent SNMV-CNN is initiated. The *Z*_*m*_ and *d*_*m*_ can be mathematically expressed as shown in equations (7) and (8), respectively.

(7) $$\begin{array}{c} Z_{m}=\sum_{1}^{n}D_{m}\left(i\right)\exp\left(-d_{m}\times c_{i}\times f_{m}\left(x_{i}\right)\right), \end{array}$$

(8) $$\begin{array}{c} d_{m}=\frac{1}{2}\ln\left(\frac{1-\varepsilon_{m}}{\varepsilon_{m}}\right). \end{array}$$

The ensemble model is formed of *M* weak classifiers after *m* iterations. As shown in equation (9), the final result of AdaBoost-SNMV-CNN is the combination of classification results weighted by *d*_*m*_:

(9) $$\begin{array}{c} F\left(x\right)=\text{sign}\left(\sum_{1}^{M}d_{m}\times f_{m}\left(x\right)\right). \end{array}$$

Finally, by using weighted or simple voting schemes, the constructed individual classifiers were combined to form the composite classifier.

**Figure 3:**
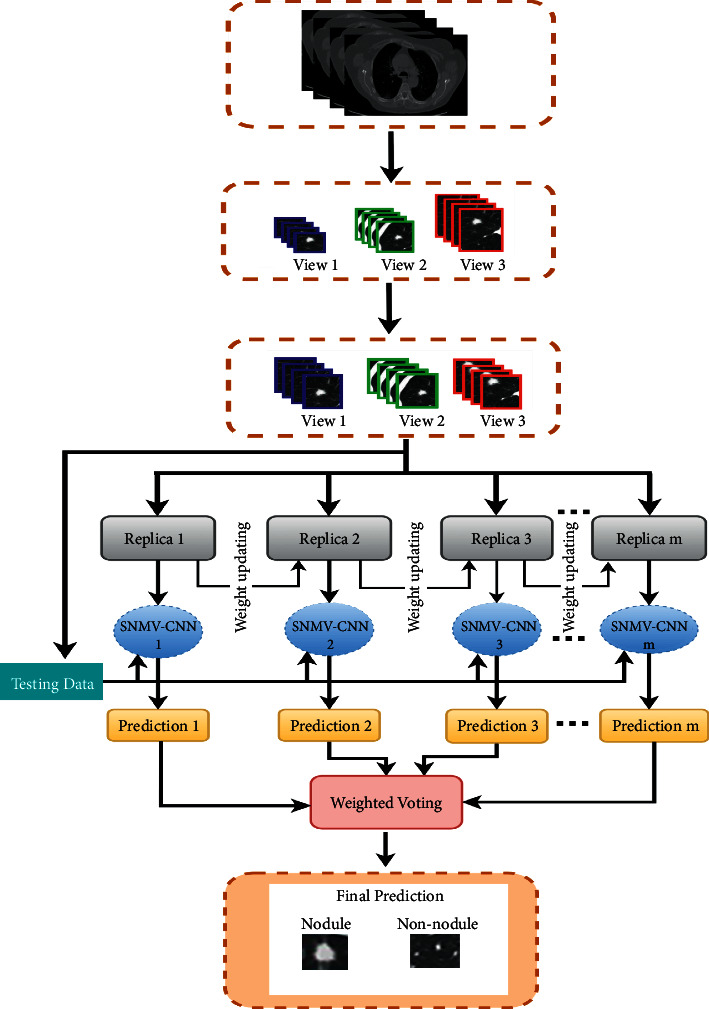
Schematic of the proposed AdaBoost-SNMV-CNN. In the preprocessing stage, lung cancer image data were divided into training (80%) and testing (20%) subsets. To train the AdaBoost-SNMV-CNN, the data weight was initialized. The first SNMV-CNN was trained using the initial data weight, then SNMV-CNN was used to update the data weight of the second SNMV-CNN. This process continues until the *M*^*th*^ SNMV-CNN was trained. Finally, the SNMV-CNN predictions for the testing subsets were combined using a weighted voting approach to get results (such as nodules or nonnodule).

The schematic diagram of the proposed AdaBoost-SNMV-CNN is shown in Figure 3. In the preprocessing stage, lung cancer image data were divided into training (80%) and testing (20%) subsets. To train the AdaBoost-SNMV-CNN, the data weight was initialized. The first SNMV-CNN was trained using the initial data weight, then SNMV-CNN was used to update the data weight of the second SNMV-CNN. This process continues until the *M*^*th*^ SNMV-CNN was trained. Finally, the SNMV-CNN predictions for the testing subsets were combined using a weighted voting approach to get results (such as nodules or nonnodule).

## 3. Results

In this section, we compare the performance of AdaBoost-SNMV-CNN with other state-of-the-art algorithms. The proposed method was implemented using Keras [28], and a high-levelopen-source library with TensorFlow [10]. The simulation was run on an Intel(R) Core(TM) i7-8750H CPU (Dual Core processor frequency: 2.2 GHz), with 32 GB of installed memory (RAM), on a 64-bit operating system. A NVIDIA GeForce GTX 1070 with a MAX-Q design GPU had been used to train the model.

### 3.1. Performance Metrics

To check the proposed model for its ability to distinguish between nodules and nonnodules, we measured the confusion matrix derived from four matrices (Table 1. The four metrics of the confusion matrix are as follows: (1) true positives (TP), (2) true negatives (TN), (3) false negatives (FN), and (4) false positives. TP indicates the number of nodules correctly detected as nodules, and TN indicates the number of nonnodules correctly detected as nonnodules. FN indicates the number of nodules incorrectly detected as nonnodule and FP indicates the number of nonnodules incorrectly detected as nodules. By using the value of the aforementioned four metrics, we further evaluated the following performance metrics for AdaBoost-SNMV-CNN.


(11) $$\begin{array}{c} \text{Sensitivity}=\frac{\text{TP}}{\text{TP}+\text{FN}}. \end{array}$$

A very straightforward metric for model evaluations is accuracy, but for its evaluation and usefulness, the model must be trained on balanced data. Any deep learning model trained on imbalanced data even with high accuracy is still less valuable [29].

(10) $$\begin{array}{c} \text{Accuracy}=\frac{\text{TP}+\text{TN}}{\text{TP}+\text{TN}+\text{FP}+\text{FN}}. \end{array}$$

Sensitivity (also called recall or true positive rate) is a useful measure for model evaluation when FN has a high cost. The higher the sensitivity, the lower the probability of missing nodules. Sensitivity is computed as follows.

Specificity is the fraction of true negative samples identified by the model. Specificity is measured as follows:

(12) $$\begin{array}{c} \text{Specificity}=\frac{\text{TN}}{\text{TN}+\text{FP}}. \end{array}$$

Additionally, we define the true positive rate (TPR) and false positive rate (FPR) as described in equations (13) and (14), respectively. The TPR and FPR are used to plot the receiver operating characteristic (ROC) curve, which was used to evaluate the trained models on both datasets [30]. The area under the receiver operating characteristic curve (AUROCC) is a frequently used metric for assessing a model's ability to discriminate across classes. A model with a higher AUROCC is generally considered to be more useful for distinguishing between classes (such as nodules and nonnodules).

(13) $$\begin{array}{c} \text{TPR}=\frac{\text{TP}}{\text{TP}+\text{FN}}, \end{array}$$

(14) $$\begin{array}{c} \text{FPR}=\frac{\text{FP}}{\text{TN}+\text{FP}}. \end{array}$$

### 3.2. Comparison of AdaBoost-SNMV-CNN Performance with Other State-of-the-Art Methods

In this section, we compared the performance of the proposed AdaBoost-SNMV-CNN with state-of-the-art methods.

#### 3.2.1. Performance of AdaBoost-SNMV-CNN on the LIDC-IDRI Dataset

In order to validate the effectiveness of the network model, the accuracy, sensitivity, specificity, and receiver operating characteristic (ROC) curve were evaluated.

**Figure 4:**
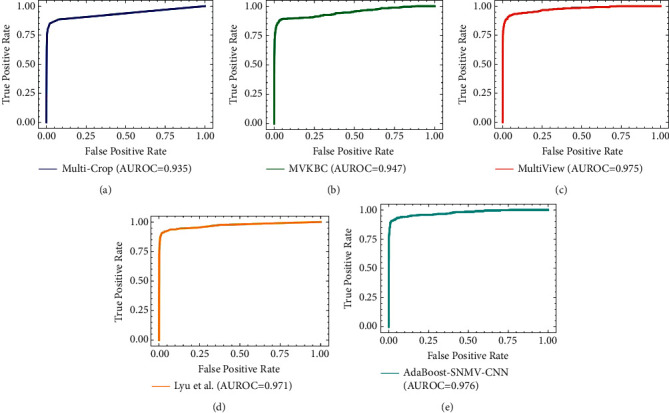
Comparison of the proposed method with other state-of-the-art methods in terms of AUROC values. AUROC of (a) multicrop method, (b) MVKBC method, (c) multiview method, (d) Lyu et al. method, and (e) proposed AdaBoost-SNMV-CNN.

The experiments were performed using 10-foldcross-validation, and the validation dataset and test dataset were swapped to repeat the experiment. The average results were taken as the final experiment result. To confirm the effectiveness of the AdaBoost-SNMV-CNN, the hyperparameters of the ADAM optimizer momentum were set to: 0.9 and learning rate (LR) = 0.0001. Because the model requires that the input size of the image should be 224 × 224 × 1, the extracted multiple-view images were resized to the same size before being input into AdaBoost-SNMV-CNN. In order to evaluate the effectiveness of AdaBoost-SNMV-CNN, we compared its performance with five different models published before; the results are tabulated in Table 2. As mentioned, the results were obtained based on 10-foldcross-validation. The deep-learning-basedtwo-stage CNN model achieves an accuracy of 84.40%, sensitivity of 83.55%, and specificity of 91.59% [31]. Similarly, the multicrop achieves an accuracy of 87.14%, sensitivity of 77%, and specificity of 93% [32]. Xie et al. [33] proposed a multiview knowledge-based collaborative (MVKBC) deep learning model. The proposed model obtained a sensitivity, specificity, accuracy, and AUC score of 86.52%, 94.00%, 91.60%, and 95.70%. Zhao et al. [34] introduced a new Agile convolutional neural network (CNN) framework for nodules identification and achieved an accuracy of 82.23% with an AUROC value of 0.877. Lyu et al. [35] introduced a multilevel cross-residual-based CNN for lung nodules identification; achieved a sensitivity, specificity, accuracy, and AUROC of 92.10%, 91.50%, 92.19%, and 97.05% on the LIDC-IDRI dataset. Given these experimental results, we can see that the proposed AdaBoost-SNMV-CNN network achieved better results for the identification of nodules from nonnodules. Table 2 shows that the accuracy (92%), sensitivity (93%), and specificity (92%) of the proposed AdaBoost-SNMV-CNN method for lung cancer nodule detection are higher than that of the existing methods. We also plot the AUROC curve of the proposed method and other existing methods as shown in Figure 4: (a) is the AUROC curve (0.935) of the multicrop method, (b) is the AUROC curve (0.94) of the MVKBC, (c) represents the AUROC curve (0.975) of the multiview method, (d) represents the AUROC curve (0.971) of the Lyu et al. method, and (e) represents the AUROC curve (0.976) of the AdaBoost-SNMV-CNN. The proposed method improvised better AUROC value than the existing method proving its prowess for better identification of nodules from nonnodules.

#### 3.2.2. Performance of AdaBoost-SNMV-CNN on the ELCAP Dataset

**Figure 5:**
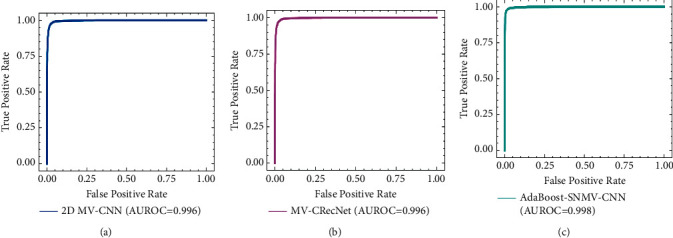
Comparison of the proposed method AUROCC value with existing methods on ELCAP dataset. The AUROC of (a) 2D MV-CNN, (b) MV-CRecNET, and (c) Proposed AdaBoost-SNMV-CNN.

For training the proposed model on the ELCAP dataset, nodules were from the ELCAP dataset, whereas nonnodules were taken from LIDC-IDRI dataset. On the ELCAP dataset, it can be observed that 2D CNNs achieved very high values for all evaluation metrics (accuracy, sensitivity, and specificity). This may be due to the fact that the ELCAP dataset consists of low-dose CT scans, whereas the LIDC-IDRI dataset contains normal/high-dose CT scans. It may therefore be the case that, while training the models on different dose CT scans, the models may have learned the biases of both datasets to correctly classify the majority of examples. The model might be learning that if a normal/high-dose CT scan is presented, then it constitutes a negative example and classifies it accordingly, whereas if a low-dose CT scan is presented, then it is treated as a positive example and classified as a nodule. From Table 3, we can observe that the 2D CNNs model achieved good performance, but it has lower sensitivity compared to other methods [21]. This may be because its receptive field is not able to cover a sufficient amount of contextual information for nodules; some nodules were large and did not fit into the model's receptive field. The reason for this could be that its receptive field not only covered nonnodules correctly but also nodule locations. In order to combine the benefits of different-sized receptive fields, we implemented the AdaBoost-SNMV-CNN model. Results reveal that AdaBoost-SNMV-CNN obtained the highest values, among the 2D MV-CNN models, for accuracy, specificity, sensitivity, and AUC. From Table 3 and Figure 5, it can be observed that the proposed method in terms of accuracy, sensitivity, and specificity achieved similar results as reported in [21].

### 3.3. Training Time of AdaBoost-SNMV-CNN

We noted that the training time of AdaBoost-SNMV-CNN is ≈10^2^ min. There are three reasons for the fast training time of the designed AdaBoost-SNMV-CNN. Reason number 1 is its simple architecture compared to other methods, as shown in Table 2. Reason number 2 is the property of weight updating during the training stage. Reason number 3 is the AdaBoost-SNMV-CNN self-normalization ability to solve the problem of gradient disappearance and explosion in the training phase. For model optimization experiments, a first-ordergradient-based optimization algorithm called Adaptive Moments (ADAM) was used having a learning rate of 0.0001, a loss function was a binary cross-entropy, and a batch size of 128. For evaluating the training time of the proposed method, a built-in python library (https://docs.python.org/3/library/time.html) was used. The final value of the training time was the average of the 10 repetitions. Therefore, it is reasonable to state that the AdaBoost-SNMV-CNN method needs less time for training, while simultaneously achieving higher accuracy.

## 4. Discussion

Medical imaging has been considered a valuable tool in clinical oncology and is favored for its noninvasive nature. CT images are most commonly used in designing computer-aided diagnostic tools for the detection of lung nodules [36]. CT imaging has advantages such as cost effectiveness, wider scale availability, high sensitivity, and fast acquisition. As lung cancer's early representation can be in the form of nodules in the lung, nodules detection is vital for the early diagnosis of lung cancer. One major problem in nodules detection is the high similarity of false nodules candidates (nonnodules) with true nodules in terms of intensity and morphology. Therefore, it is a repetitive and arduous task for radiologists to identify all suspicious nodules from nonnodules through CT imaging. Hence, the development of an accurate, sensitive, and specific computer-aided detection system is pertinent [37]. The proposed AdaBoost-SNMV-CNN achieved an accuracy of 92%, sensitivity of 93%, and specificity of 92% for lung nodules detection on the LIDC-IDRI dataset. Meanwhile, on the ELCAP dataset, the accuracy for detecting lung nodules was 99%, sensitivity 100%, and specificity 98%. AdaBoost-SNMV-CNN outperformed the majority of models in accuracy, sensitivity, and specificity. The multiviews confer the model's good generalization and learning ability for diverse features of lung nodules, and the model architecture is simple and has a minimal computational time of around 10^2^ minutes. A group of researchers used 3D CNN and SVM to construct a fusion model for assessing the malignancy of lung nodules using the LIDC-IDRI data [38]. The method they proposed achieved an accuracy of 85%. Using the LUNA16 dataset, a two-stage CNN network including two CNNs was proposed. The first stage includes image refinement followed by the second stage Google Nets to accomplish classification with around 89.6% accuracy [9]. A model using 11 deep CNNs and transfer learning has achieved an accuracy of 88% [39]. Transfer learning has been proved to be effective in designing deep learning-based methods for medical purposes [40]. The proposed AdaBoost-SNMV-CNN utilizes transfer learning as well. A deep learning-based CAD for nodules detection using the Chinese LDCT dataset. The method achieved a sensitivity of 90% compared to the sensitivity achieved by the double reading of 76%. They conclude that the method can assist radiologists in decision-making. Similarly, our method achieved a sensitivity of 93% on the LIDC-IDRI dataset and about 100% sensitivity on the ELCAP dataset. Encouraged by the conclusion of the aforementioned study, we envision testing our method with the double reading to establish the feasibility of our method as an alternative for assisting the radiologist [41]. Xie et al. developed a method to quickly read and locate the lung nodules by using 2D CNN. They achieved a sensitivity of 86.42% and an accuracy of 88.8% [42]. An Inverse Surface Adaptive Thresholding (ISAT)- and Artificial Neural Network-based platforms have achieved an accuracy of 90.0% for classifying between nodules' nature whether cancerous or benign. A group of researchers tried to optimize the traditional R-CNN and Faster R-CNN and improved the lung cancer nodules detection accuracy by 20%. The accuracy of the original Faster R-CNN was 82.4%, optimized and improved Faster R-CNN was 91.2%, R-CNN 68.4%, and Fast R-CNN 75.4%. Compared to the above-mentioned methods, the proposed AdaBoost-SNMV-CNN has an accuracy of 92% on the LIDC-IDRI dataset and 99% accuracy on the ELCAP dataset [43].

## 5. Limitations and Future Work

As our method has shown considerable success in identifying the lung cancer nodules in CT images, it offers a good alternative to eliminate issues such as misdiagnosis and missed diagnosis of lung cancer nodules. The multiple views confer AdaBoost-SNMV-CNN to effectively analyze the characteristics of lung nodules, thus some less obvious nodules caused by radiological heterogeneity can also be successfully identified as well. There are still some limitations that need to be addressed in this study. First, a complete lung nodule is often distributed on multiple slices. However, our AdaBoost-SNMV-CNN method is limited in capturing the contextual information between slices, thus a 3D CNN will be integrated into AdaBoost and may be considered in our future work. Second, our model is trained on LIDC-IDRI and ELCAP datasets. Some types of nodules are not fully represented or not fully highlighted in these datasets, which may lead to the false identification of nodules. We believe that a larger dataset for sample preparation and effective sample preprocessing before training will help identify these candidates, which will also be part of our future research work. Third some studies report that CNN is not an optimum comparison to the human visual system. In contrast, a capsule neural network is considered to be the best mimic of the human visual system. Concurrently, capsule neural networks can train on far less data and improvise more accurate results [19]. In the future, we are looking to collaborate with the hospital to acquire fresh CT images to test and train our model in order to further improve the model performance.

## 6. Conclusion

The overwhelming burden of lung cancer in terms of deaths and new cases is alarming. Lung cancer manifest in the form of lung nodules. CT scan is the most widely employed technique for the detection of nodules in the lung. However, the examination of the CT scan data is a sophisticated and laborious task for doctors/radiologists. To assist doctors/radiologists, CAD platforms can be highly valuable for the automated detection of lung nodules. The proposed AdaBoost-SNMV-CNN was trained and tested on the widely used Lung Image Database Consortium and Image Database Resource Initiative (LIDC-IDRI) and Early Lung Cancer Action Program (ELCAP) datasets. AdaBoost-SNMV-CNN achieved an accuracy of 92%, sensitivity of 93%, and specificity of 92% for lung nodules detection on the LIDC-IDRI dataset. Meanwhile, on the ELCAP dataset, the accuracy for detecting lung nodules was 99%, sensitivity 100%, and specificity 98%. AdaBoost-SNMV-CNN outperformed the majority of the model in accuracy, sensitivity, and specificity. The multiviews confer the model's good generalization and learning ability for diverse features of lung nodules, and the model architecture is simple and has a minimal computational time of around 10^2^ minutes. The incorporation of multiview strategy supports the model in learning the most important and critical features that can be generalized to improve the model performance and achieve state-of-the-art results for lung cancer nodules identification. We anticipate that the model has the capability to be tested across other medical imaging-based diagnostics for a variety of cancers. Further testing across multiple-imaging platforms and evaluation by an experienced radiologist are highly recommended to be able to take full advantage of the proposed method in clinical setups.

## Figures and Tables

**Algorithm 1:**
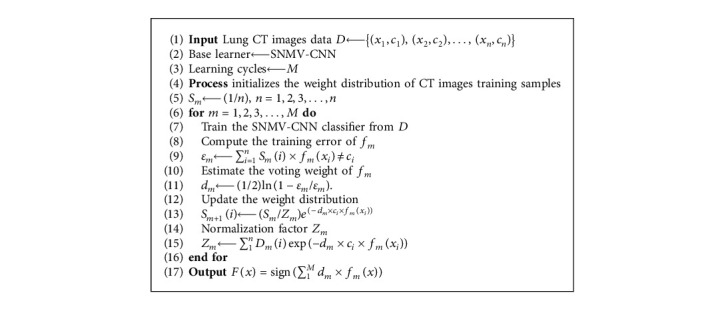
The proposed AdaBoost-SNMV-CNN.

**Table 1:** The binary confusion matrix for lung cancer nodules and nonnodules identification. The predicted labels are shown in rows while actual/true labels are in the form of columns.

	Nodule	Nonnodule
Nodule	TP (true positive)	FP (false positive)
Nonnodule	FN (false negative)	TN (true negative)

**Table 2:** Comparison of proposed AdaBoost-SNMV-CNN with different models. We can observe that the proposed method has better performance compared to other methods on the LIDC-IDRI dataset.

Dataset	Models	Accuracy	Sensitivity	Specificity
LIDC-IDRI	Two-stage CNN	0.844	0.835	0.915
	Multicrop	0.8714	0.77	0.93
	MVKBC	0.916	0.865	0.940
	Zhao et al.	0.823	—	—
	Lyu et al.	0.921	0.921	0.915
	AdaBoost-SNMV-CNN	0.929	0.930	0.921

**Table 3:** In comparison of the proposed AdaBoost-SNMV-CNN with different models, we can observe that the proposed method has a better performance compared to other methods on the ELCAP dataset.

Dataset	Models	Accuracy	Sensitivity	Specificity
ELCAP	2D MV-CNN	0.986	0.978	0.995
	MV-CRecNet	0.994	1.00	0.989
	AdaBoost-SNMV-CNN	0.999	1.000	0.988

## Data Availability

The research has been conducted using a publicly available dataset. Lung Image Database Consortium and Image Database Resource Initiative (LIDC-IDRI) can be assessed online at https://wiki.cancerimagingarchive.net/display/public/LIDC-IDRI and the Early lung cancer Action Program (ELCAP) dataset can be assessed at https://www.via.cornell.edu/lungdb.html.
